# BayesHammer: Bayesian clustering for error correction in single-cell sequencing

**DOI:** 10.1186/1471-2164-14-S1-S7

**Published:** 2013-01-21

**Authors:** Sergey I Nikolenko, Anton I Korobeynikov, Max A Alekseyev

**Affiliations:** 1 Algorithmic Biology Laboratory, Academic University, St. Petersburg, Russia; 2 St. Petersburg State University, Russia; 3 Department of Computer Science and Engineering, University of South Carolina, Columbia, SC, USA

## Abstract

Error correction of sequenced reads remains a difficult task, especially in single-cell sequencing projects with extremely non-uniform coverage. While existing error correction tools designed for standard (multi-cell) sequencing data usually come up short in single-cell sequencing projects, algorithms actually used for single-cell error correction have been so far very simplistic.

We introduce several novel algorithms based on Hamming graphs and Bayesian subclustering in our new error correction tool BAYESHAMMER. While BAYESHAMMER was designed for single-cell sequencing, we demonstrate that it also improves on existing error correction tools for multi-cell sequencing data while working much faster on real-life datasets. We benchmark BAYESHAMMER on both *k*-mer counts and actual assembly results with the SPADES genome assembler.

## Background

Single-cell sequencing [1,2] based on the Multiple Displacement Amplification (MDA) technology [1,3] allows one to sequence genomes of important uncultivated bacteria that until recently had been viewed as unamenable to genome sequencing. Existing metagenomic approaches (aimed at genes rather than genomes) are clearly limited for studies of such bacteria despite the fact that they represent the majority of species in such important studies as the Human Microbiome Project [4,5] or discovery of new antibiotics-producing bacteria [6].

**Figure 1:**
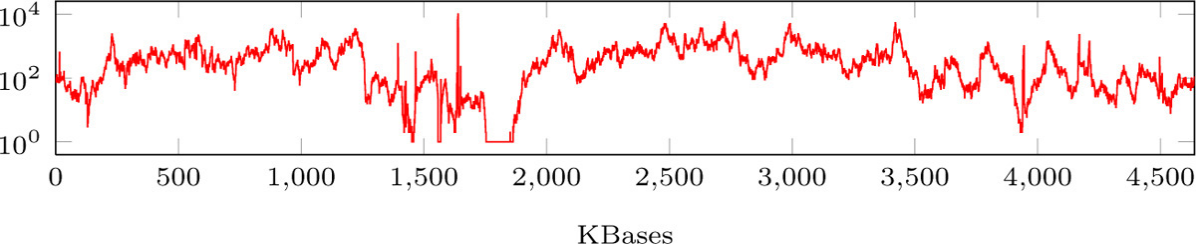
**Logarithmic coverage plot for the single-cell *E. coli *dataset**. Logarithmic coverage plot for the single-cell *E. coli *dataset (similar plot is also given in [2]).

Single-cell sequencing datasets have extremely non-uniform coverage that may vary from ones to thousands along a single genome (Figure 1). For many existing error correction tools, most notably QUAKE [7], uniform coverage is a prerequisite: in the case of non-uniform coverage they either do not work or produce poor results.

Error correction tools usually attempt to correct the set of *k*-character substrings of reads called *k-mers *and then propagate corrections to whole reads which are important to have for many assemblers. Error correction tools often employ a simple idea of discarding rare *k*-mers, which obviously does not work in the case of non-uniform coverage.

Medvedev *et al. *[8] recently presented a new approach to error correction for datasets with non-uniform coverage. Their algorithm HAMMER makes use of the Hamming graph (hence the name) on *k*-mers (vertices of the graph correspond to *k*-mers and edges connect pairs of *k*-mers with Hamming distance not exceeding a certain threshold). HAMMER employs a simple and fast clustering technique based on selecting a *central k-mer *in each connected component of the Hamming graph. Such central *k*-mers are assumed to be error-free (i.e., they are assumed to actually appear in the genome), while the other *k*-mers from connected components are assumed to be erroneous instances of the corresponding central *k*-mers. However, HAMMER may be overly simplistic: in connected components of large diameter or connected components with several *k*-mers of large multiplicities, it is more reasonable to assume that there are two or more central *k*-mers (rather than one as in HAMMER). Biologically, such connected components may correspond to either (1) repeated regions with similar but not identical genomic sequences (*repeats*) which would be bundled together by existing error correction tools (including HAMMER); or (2) artificially united *k*-mers from distinct parts of the genome that just happen to be connected by a path in the Hamming graph (characteristic to HAMMER).

In this paper, we introduce the BAYESHAMMER error correction tool that does not rely on uniform coverage. BAYESHAMMER uses the clustering algorithm of HAMMER as a first step and then refines the constructed clusters by further subclustering them with a procedure that takes into account reads quality values (e.g., provided by Illumina sequencing machines) and introduces Bayesian (BIC) penalties for extra subclustering parameters. BAYESHAMMER subclustering aims to capture the complex structure of repeats (possibly of varying coverage) in the genome by separating even very similar *k*-mers that come from different instances of a repeat. BAYESHAMMER also uses a new approach for propagating corrections in *k*-mers to corrections in the reads. All algorithms in BAYESHAMMER are heavily parallelized whenever possible; as a result, BAYESHAMMER gains a significant speedup with more processing cores available. These features make BAYESHAMMER a perfect error correction tool for single-cell sequencing.

We remark that HAMMER produces only a set of central *k*-mers but does not correct reads, making it incompatible with most genome assemblers. QUAKE does correct reads but has severe memory limitations for large *k *and assumes uniform coverage. In contrast, EULER-SR [9] and CAMEL [2] correct reads and do not make strong assumptions on coverage (both tools have been used for single-cell assembly projects [2]) which makes these tools suitable for comparison to BAYESHAMMER. Our benchmarks show that BAYESHAMMER outperforms these tools in both single-cell and standard (multi-cell) modes. We further couple BAYESHAMMER with a recently developed genome assembler SPADES [10] and demonstrate that assembly of BAYESHAMMER-corrected reads significantly improves upon assembly with reads corrected by other tools for the same datasets, while the total running time also improves significantly.

BAYESHAMMER is freely available for download as part of the SPADES genome assembler at http://bioinf.spbau.ru/spades/.

## Methods

### Notation and outline

Let ∑ = {*A, C, G, T*} be the alphabet of nucleotides (BAYESHAMMER discards *k*-mers with uncertain bases denoted *N*). A *k*-mer is an element of ∑*^k^*, i.e., a string of *k *nucleotides. We denote the *i*^th ^letter (nucleotide) of a *k*-mer *x *by *x*[*i*], indexing them from zero: 0 ≤ *i *≤ *k *- 1. A subsequence of *x *corresponding to a set of indices *I *is denoted by *x*[*I*]. We use interval notation [*i, j*] for intervals of integers {*i, i *+ 1,..., *j*} and further abbreviate *x*[*i, j*] = *x *[{*i, i *+ 1,..., *j*}]; thus, *x *= *x*[0, *k *- 1]. Input reads are represented as a set of strings *R *⊂ Σ* along with their *quality values *${\left(q_{r}\left[i\right]\right)}_{i=0}^{|r|-1}$ for each *r *∈ *R*. We assume that *q_r_*[*i*] estimates the probability that there has been an error in position *i *of read *r*. Notice that in practice, the fastq file format [11] contains characters that encode probabilities on a logarithmic scale (in particular, products of probabilities used below correspond to sums of actual quality values).

**Figure 2:**
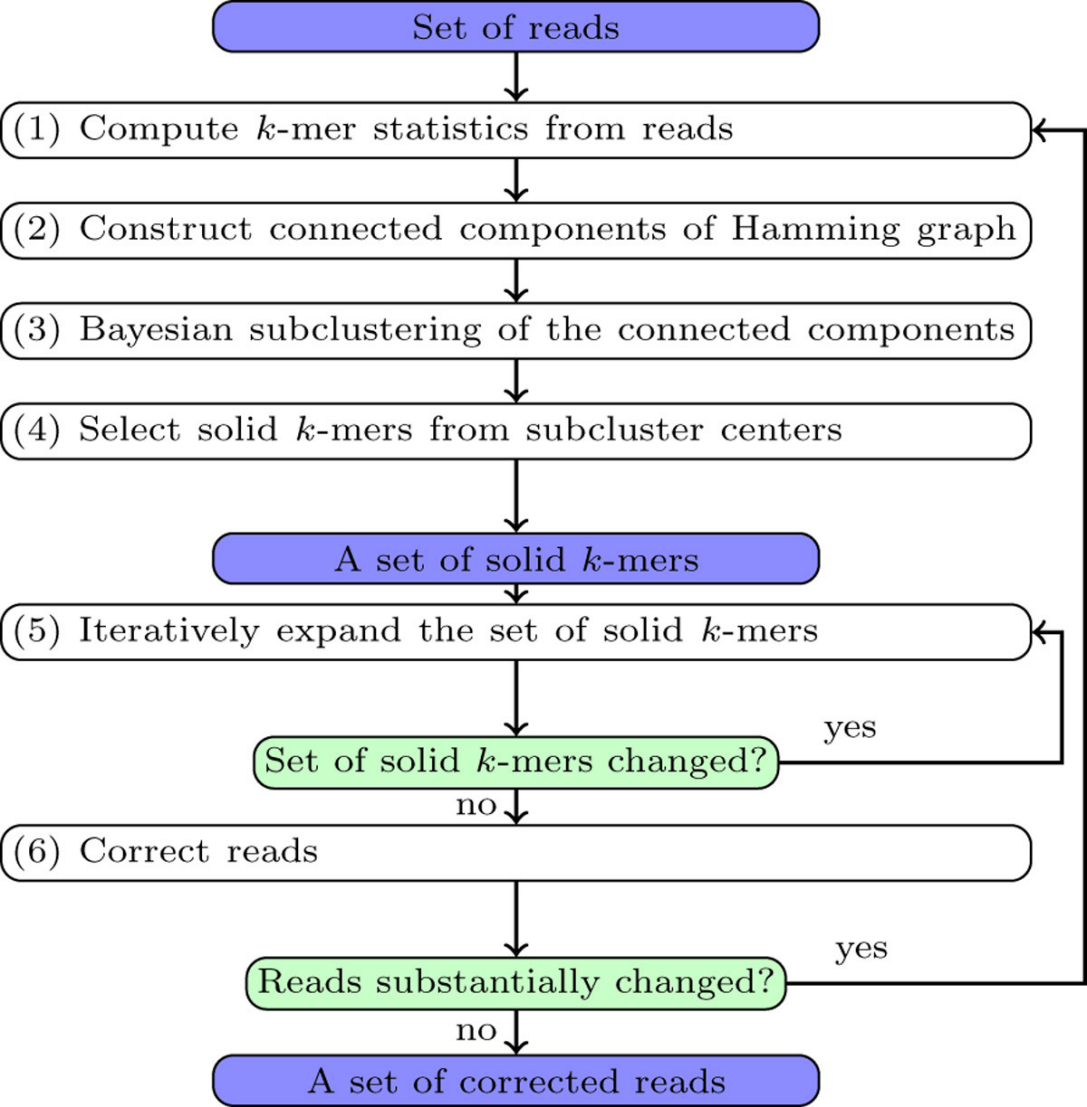
**BAYESHAMMER workflow**.

Below we give an overview of BAYESHAMMER workflow (Figure 2) and refer to subsequent sections for further details. On Step (1), *k*-mers in the reads are counted, producing a triple *statistics*(*x*) = (*count_x_, quality_x_*, **error***_x_*) for each *k*-mer *x*. Here, *count_x _*is the number of times *x *appears as a substring in the reads, *quality_x _*is its total quality expressed as a probability of sequencing error in *x*, and **error***_x _*is a *k*-dimensional vector that contains products of error probabilities (sums of quality values) for individual nucleotides of *x *across all its occurrences in the reads. On Step (2), we find connected components of the Hamming graph constructed from this set of *k*-mers. On Step (3), the connected components become subject to Bayesian subclustering; as a result, for each *k*-mer we know the center of its subcluster. On Step (4), we filter subcluster centers according to their total quality and form a set of *solid k*-mers which is then iteratively expanded on Step (5) by mapping them back to the reads. Step (6) deals with reads correction by counting the majority vote of solid *k*-mers in each read. In the iterative version, if there has been a substantial amount of changes in the reads, we run the next iteration of error correction; otherwise, output the corrected reads. Below we describe specific algorithms employed in the BAYESHAMMER pipeline.

### Algorithms

#### Step (1): computing k-mer statistics

To collect *k*-mer statistics, we use a straightforward hash map approach [12] that does not require storing instances of all *k*-mers in memory (as excessive amount of RAM might be needed otherwise). For a certain positive integer *N *(the number of auxiliary files), we use a hash function *h*: ∑*^k ^*→ℤ*_N _*that maps *k*-mers over the alphabet Σ to integers from 0 to *N *- 1.

**Algorithm 1 **Count *k*-mers

**for **each *k*-mer *x *from the reads *R*: **do**

compute *h*(*x*) and write *x *to File_*h*(*x*)_.

**for ***i *∈ [0, *N ***- **1]: **do**

sort File*_i _*with respect to the lexicographic order;

reading File*_i _*sequentially, compute *statistics*(*s*) for each *k*-mer *s *from File*_i_*.

#### Step (2): constructing connected components of Hamming graph

Step (2) is the essence of the HAMMER approach [8]. The *Hamming distance *between *k*-mers *x, y *∈ ∑*^k ^*is the number of nucleotides in which they differ:

$$\text{d}\left(x,y\right)=\left|\left\{i\in \left[0,k-1\right]:x\left[i\right]\neq y\left[i\right]\right\}\right|.$$

**Figure 3:**
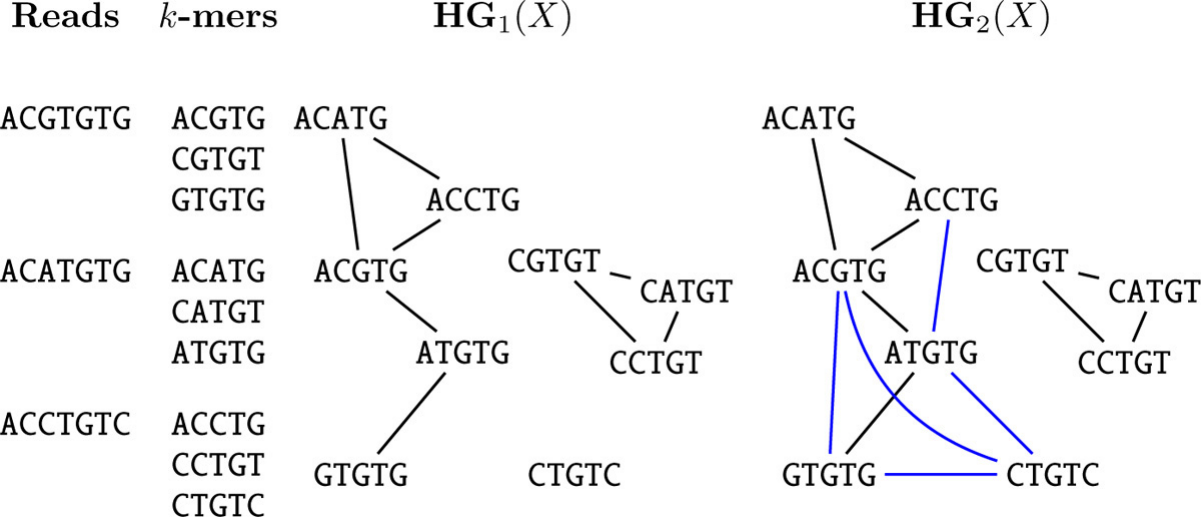
**Hamming graphs HG_1_(*X*) and HG_2_(*X*)**. Hamming graphs HG_1_(*X*) and HG_2_(*X*) for *X *being the set of 4-mers {ACGTG, CGTGT, GTGTG, ACATG, CATGT, ATGTG, ACCTG, CCTGT, CTGTC} of the reads ACGTGTG, ACATGTG, ACCTGTC. Blue edges denote Hamming distance 2.

For a set of *k*-mers *X*, the *Hamming graph *HG*_τ_*(X) is an undirected graph with the set of vertices *X *and edges corresponding to pairs of *k*-mers from *X *with Hamming distance at most τ, i.e., *x, y *∈ *X *are connected by an edge in HG*_τ_*(*X*) iff d(*x, y*) ≤ *τ *(Figure 3). To construct HG*_τ_*(*X*) efficiently, we notice that if two *k*-mers are at Hamming distance at most *τ*, and we partition the set of indices [0,*k *- 1] into *τ *+ 1 parts, then at least one part corresponds to the same subsequence in both *k*-mers. Below we assume with little loss of generality that *τ *+ 1 divides *k*, i.e., *k *= *σ *(*τ *+ 1) for some integer *σ*.

For a subset of indices *I *⊆ [0, *k *- 1], we define a partial lexicographic ordering ≺*_I _*as follows: *x *≺*_I _y *iff *x*[*I*] ≺ *y*[*I*], where ≺ is the lexicographic ordering on Σ*. Similarly, we define a partial equality =*_I _*such that *x *=*_I _y *iff *x*[*I*] = *y*[*I*]. We partition the set of indices [0, *k *- 1] into *τ *+ 1 parts of size *σ *and for each part *I*, sort a separate copy of *X *with respect to ≺*_I_*. As noticed above, for every two *k*-mers *x, y *∈ *X *with d(*x, y*) ≤ *τ*, there exists a part *I *such that *x *=*_I _y*. It therefore suffices to separately consider blocks of equivalent *k*-mers with respect to =*_I _*for each part *I*. If a block is small (i.e., of size smaller than a certain threshold), we go over the pairs of *k*-mers in this block to find those with Hamming distance at most *τ*. If a block is large, we recursively apply to it the same procedure with a different partition of the indices. In practice, we use two different partitions of [0, *k *- 1]: the first corresponds to contigious subsets of indices (recall that $\sigma =\frac{k}{\tau +1}$):

**Algorithm 2 **Hamming graph processing

**procedure **HGPROCESS(*X*, max_quadratic)

Init components with singletons X={{x}:x∈X}.

**for all ***ϒ *∈ FindBlocks$\left(X,{\left\{I_{s}^{\text{cnt}}\right\}}_{s=0}^{\tau }\right)$**do**

**if **|*ϒ*| > max_quadratic **then**

   **for all ***Z *∈ FindBlocks $\left(ϒ,{\left\{I_{s}^{\text{str}}\right\}}_{s=0}^{\tau }\right)$**do**

      ProcessExhaustively(Z,X)

else

   ProcessExhaustively(ϒ,X).

**function **FindBlocks $\left(X,{\left\{I_{s}\right\}}_{s=0}^{\tau }\right)$

**for ***s *= 0,...,*τ ***do**

sort a copy of *X *with respect to ${≺}_{I_{s}}$, getting *X_s_*.

**for ***s *= 0,...,*τ ***do**

output the set of equiv. blocks $\left\{ϒ\right\}\;\text{w}.\text{r}.\text{t}.{=}_{I_{s}}$.

**procedure **PROCESSEXHAUSTIVELY(ϒ,X)

**for **each pair *x, y *∈ *ϒ ***do**

**if **d(*x, y*) ≤ *τ ***then **join their sets in  X:

   **for all $x\in Z_{x}\in X,y\in Z_{y}\in X$ do**

      $X:=X\cup \left\{Z_{x}\cup Z_{y}\right\}\backslash \left\{Z_{x},Z_{y}\right\}$.

$$I_{s}^{\text{cnt}}=\left\{s\sigma ,s\sigma +1,\ldots ,s\sigma +\sigma -1\right\},\;s=0,\ldots ,\tau ,$$

while the second corresponds to strided subsets of indices:

$$I_{s}^{\text{str}}=\left\{s,s+\tau +1,s+2\left(\tau +1\right),\ldots ,s+\left(\sigma -1\right)\left(\tau +1\right)\right\},\;s=0,\ldots ,\tau .$$

**Figure 4:**
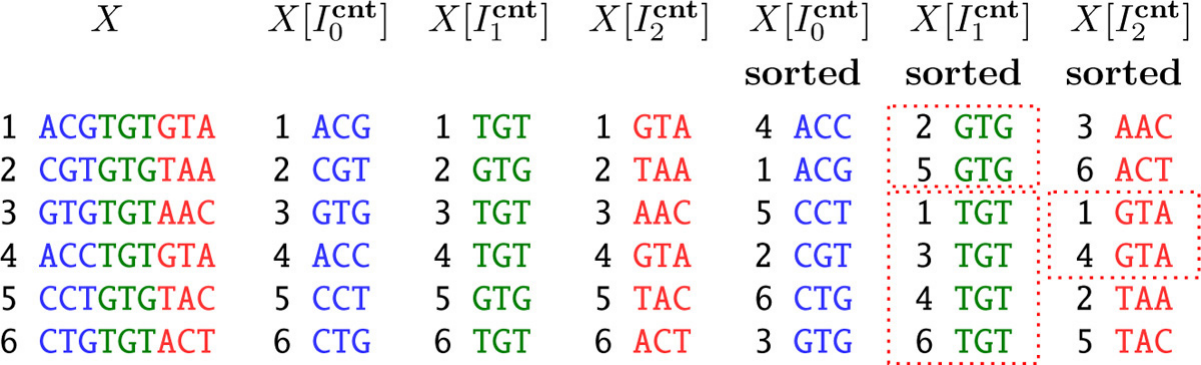
**Partial lexicographic orderings**. Partial lexicographic orderings of a set *X *of 9-mers with respect to the index sets $I_{0}^{\text{cnt}}=\left\{0,1,2\right\}$, $I_{1}^{\text{cnt}}=\left\{3,4,5\right\}$, and $I_{2}^{\text{cnt}}=\left\{6,7,8\right\}$. Red dotted lines indicate equivalence blocks.

BAYESHAMMER uses a two-step procedure, first splitting with respect to ${\left\{I_{s}^{\text{cnt}}\right\}}_{s=0}^{\tau }$ (Figure 4) and then, if an equivalence block is large, with respect to ${\left\{I_{s}^{\text{str}}\right\}}_{s=0}^{\tau }$. On the block processing step, we use the disjoint set data structure [12] to maintain the set of connected components. Step (2) is summarized in Algorithm 2.

#### Step (3): Bayesian subclustering

In HAMMER's generative model [8], it is assumed that errors in each position of a *k*-mer are independent and occur with the same probability *ε*, which is a fixed global parameter (HAMMER used *ε *= 0.01). Thus, the likelihood that a *k*-mer *x *was generated from a *k*-mer *y *under HAMMER's model equals

$$L_{{\text{H}}_{\text{AMMER}}}\left(x|y\right)={\left(1-\varepsilon \right)}^{k-d\left(x,y\right)}\varepsilon^{d\left(x,y\right)}.$$

Under this model, the maximum likelihood center of a cluster is simply its consensus string [8].

In BAYESHAMMER, we further elaborate upon HAMMER's model. Instead of a fixed ε, we use reads quality values that approximate probabilities *q_x_*[*i*] of a nucleotide at position *i *in the *k*-mer *x *being erroneous. We combine quality values from identical *k*-mers in the reads: for a multiset of *k*-mers *X *that agree on the *j*^th ^nucleotide, it is erroneous with probability Π_*x*∈*X *_*q_x_*[*j*].

The likelihood that a *k*-mer *x *has been generated from another *k*-mer *c *(under the independent errors assumption) is given by

$$L\left(x|c\right)=\underset{j:x\left[j\right]\neq c\left[j\right]}{\prod }q_{x}\left[j\right]\underset{j:x\left[j\right]=c\left[j\right]}{\prod }\left(1-q_{x}\left[j\right]\right),$$

and the likelihood of a specific subclustering *C *= *C*_1 _∪... ∪ *C_m _*is

$$L_{m}\left(C_{1},\ldots ,C_{m}\right)=\overset{m}{\underset{i=1}{\prod }}\underset{x\in C_{i}}{\prod }L\left(x|c_{i}\right)$$

where *c_i _*is the center (consensus string) of the subcluster *C_i_*.

In the subclustering procedure (see Algorithm 3), we sequentially subcluster each connected component of the Hamming graph into more and more clusters with the classical *k*-means clustering algorithm (denoted *m*-means since *k *has different meaning). For the objective function, we use the likelihood as above penalized for overfitting with the Bayesian information criterion (BIC) [13]. In this case, there are |C| observations in the dataset, and the total number of parameters is 3 *km *+ *m *- 1:

• *m *- 1 for probabilities of subclusters,

• *km *for cluster centers, and

• 2 *km *for error probabilities in each letter: there are 3 possible errors for each letter, and the probabilities should sum up to one. Here error probabilities are conditioned on the fact that an error has occurred (alternatively, we could consider the entire distribution, including the correct letter, and get 3 *km *parameters for probabilities but then there would be no need to specify cluster centers, so the total number is the same).

**Algorithm 3 **Bayesian subclustering

**for all **connected components *C *of the Hamming graph **do**

*m *:= 1

*ℓ*_1 _:= 2 log *L*_1_(*C*) (likelihood of the cluster generated by the consensus)

repeat

*m *:= *m *+ 1

do *m*-means clustering of *C *= *C*_1 _∪...∪ *C_m _*w.r.t. the Hamming distance; the initial approximation to the centers is given by *k*-mers that have the least error probability

*ℓ_m _*:= 2 · log *L_m_*(*C*_1_,...,*C_m_*) (3 *km *+ *m *- 1) · log |C|

**until ***ℓ_m _***≤ ***ℓ*_*m*-1_

output the best found clustering *C *= *C*_1 _∪...∪ *C*_*m*-1_

Therefore, the resulting objective function is

$$\ell_{m}:=2\cdot \text{log}L_{m}\left(C_{1},\ldots ,C_{m}\right)-\left(3km+m-1\right)\cdot \text{log}|C|$$

for subclustering into *m *clusters; we stop as soon as *ℓ_m _*ceases to increase.

#### Steps (4) and (5): selecting solid k-mers and expanding the set of solid k-mers

We define the quality of a *k*-mer *x *as the probability that it is error-free: $p_{x}=\prod_{j=0}^{k-1}\left(1-q_{x}\left[j\right]\right)$. The *k*-mer qualities are computed on Step (1) along with computing *k*-mer statistics. Next, we (generously) define the quality of a cluster *C *as the probability that at least one *k*-mer in *C *is correct:

$$p_{C}=1-\underset{x\in C}{\prod }\left(1-p_{x}\right).$$

In contrast to HAMMER, we do not distinguish whether the cluster is a singleton (i.e., |C| = 1); there may be plenty of superfluous clusters with several *k*-mers obtained by chance (actually, it is more likely to obtain a cluster of several *k*-mers by chance than a singleton of the same total multiplicity).

Initially we mark as *solid *the centers of the clusters whose total quality exceeds a predefined threshold (a global parameter for BAYESHAMMER, set to be rather strict). Then we expand the set of solid *k*-mers iteratively: if a read is completely covered by solid *k*-mers we conclude that it actually comes from the genome and mark all other *k*-mers in this read as solid, too (Algorithm 4).

#### Step (6): reads correction

**Figure 5:**
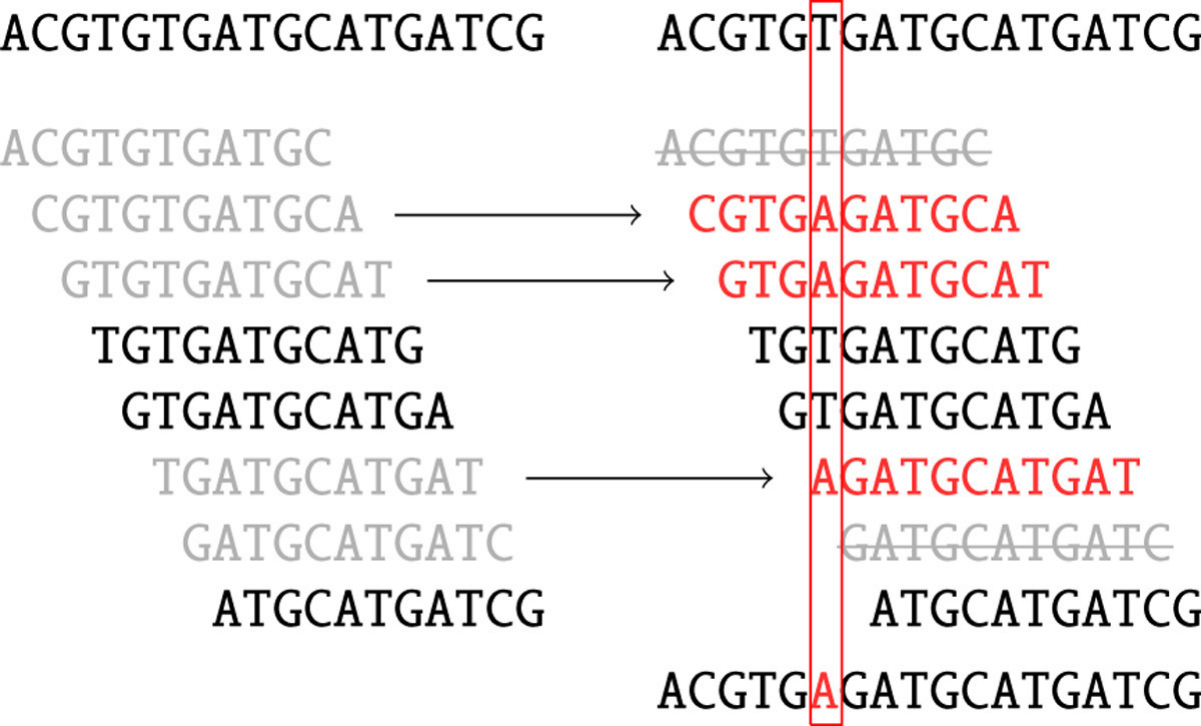
**Read correction**. Reads correction. Grey *k*-mers indicate non-solid *k*-mers. Red *k*-mers are the centers of the corresponding clusters (two grey *k*-mers striked through on the right are non-solid singletons). As a result, one nucleotide is changed.

After Steps (1)-(5), we have constructed the set of solid *k*-mers that are presumably error-free. To construct corrected reads from the set of solid *k*-mers, for each base of every read, we compute the consensus of all solid *k*-mers and solid centers of clusters of all non-solid *k*-mers covering this base (Figure 5). This step is formally described as Algorithm 5.

**Algorithm 4 **Solid *k*-mers expansion

**procedure **ITERATIVEEXPANSION(*R, X*)

**while **ExpansionStep(*R, X*) **do**

**function **EXPANSIONSTEP(*R, X*)

**for all **reads *r *∈ *R ***do**

**if ***r *is completely covered by solid *k*-mers **then**

   mark all *k*-mers in *r *as solid

Return TRUE if *X *has increased and FALSE otherwise.

**Algorithm 5 **Reads correction

**Input: **reads *R*, solid *k*-mers *X*, clusters  C.

**for all **reads *r *∈ *R ***do**

init consensus array υ: [0, |*r*| - 1] × {*A, C, G, T*} → ℕ with zeros: υ(*j, x*[*i*]):= 0 for all *i *= 0,...,|*r*| - 1 and *j *= 0,...,*k *- 1

**for ***i *= 0,...,|*r*| **- ***k ***do**

**if ***r*[*i, i *+ *k ***- **1] ∈ *X *(it is solid) **then**

   **for ***j *∈ [*i, i *+ *k ***- **1] **do**

      υ(*j, r*[*i*]):= υ(*j, r*[*i*]) + 1

**if ***r*[*i, i *+ *k ***- **1] ∈ *C *for some *C *∈  C**then**

   let *x *be the center of *C*

   **if ***x *∈ *X *(*r *belongs to a cluster with solid center) **then**

      **for ***j *∈ [*i, i *+ *k ***- **1] **do**

         υ(*j, x*[*i*]):= υ(*j, x*[*i*]) + 1

**for ***i *∈ [0, |*r*| **- **1] **do**

*r*[*i*]:= arg max_*a*∈Σ _υ(*i, a*).

## Results and discussion

### Datasets

In our experiments, we used three datasets from [2]: a single-cell *E. coli*, a single-cell *S. aureus*, and a standard (multicell) *E. coli *dataset. Paired-end libraries were generated by an Illumina Genome Analyzer IIx from MDA-amplified single-cell DNA and from multicell genomic DNA prepared from cultured *E. coli*, respectively These datasets consist of 100 bp paired-end reads with insert size 220; both *E. coli *datasets have average coverage ≈ 600×, although the coverage is highly non-uniform in the single-cell case.

In all experiments, BAYESHAMMER used *k *= 21 (we observed no improvements for higher values of *k*).

### *k*-mer counts

Table 1 shows error correction statistics produced by di erent tools on all three datasets. For a comparison with HAMMER, we have emulated HAMMER with read correction by turning off Bayesian subclustering (*HammerExpanded *in the table) and both Bayesian subclustering and read expansion, another new idea of BAYESHAMMER (*HammerNoExpansion *in the table). Note that despite its more complex processing, BAYESHAMMER is significantly faster than other error correction tools (except, of course, for HAMMER which is a strict subset of BAYESHAMMER processing in our experiments and is run on BAYESHAMMER code). BAYESHAMMER also produces, in the single-cell case, a much smaller set of *k*-mers in the resulting reads which leads to smaller de Bruijn graphs and thus reduces the total assembly running time. Since BAYESHAMMER trims only bad quality bases and does not, like QUAKE, trim bases that it has not been able to correct (it has been proven detrimental for single-cell assembly in our experiments), it does produce a much larger set of *k*-mers than Quake on a multi-cell dataset.

**Table 1:** *k*-mer statistics.

Correction tool	Running time	*k*-mers					Reads
		**Total**	**Genomic**	**Non-genomic**	**% of all genomic *k*-mers found in reads**	**% genomic among all *k*-mers in reads**	**% reads aligned to genome**

		**Multi-cell *E. coli***, total 4,543,849 genomic *k*-mers					

Uncorrected		187,580,875	4,543,684	183,037,191	99.99	2.4	99.05
Quake		4,565,237	4,543,461	21,776	99.99	99.5	99.97
HammerNoExpansion	30 m	58,305,738	4,543,674	53,762,064	99.99	8.4	95.59
HammerExpanded	36 m	28,290,788	4,543,673	23,747,115	99.99	19.1	99.49
BayesHammer	37 m	27,100,305	4,543,674	22,556,631	99.99	20.1	99.62

		**Single-cell *E. coli***, total 4,543,849 genomic *k*-mers					

Uncorrected		165,355,467	4,450,489	160,904,978	97.9	2.7	79.05
Camel	2 h 29 m	147,297,070	4,450,311	142,846,759	97.9	3.0	81.25
Euler-SR	2 h 15 m	138,677,818	4,450,431	134,227,387	97.9	3.2	81.95
Coral	2 h 47 m	156,907,496	4,449,560	152,457,936	97.9	2.8	80.28
HammerNoExpansion	37 m	53,001,778	4,443,538	48,558,240	97.8	8.3	81.36
HammerExpanded	43 m	36,471,268	4,443,545	32,027,723	97.8	12.1	86.91
BayesHammer	57 m	35,862,329	4,443,736	31,418,593	97.8	12.4	87.12

		**Single-cell *S. aureus***, total 2,821,095 genomic *k*-mers					

Uncorrected		88,331,311	2,820,394	85,510,917	99.98	3.2	75.07
Camel	5 h 13 m	69,365,311	2,820,350	66,544,961	99.97	4.1	75.27
Euler-SR	2 h 33 m	58,886,372	2,820,349	56,066,023	99.97	4.8	75.24
Coral	7 h 12 m	83,249,146	2,820,011	80,429,135	99.96	3.4	75.22
HammerNoExpansion	58 m	37,465,296	2,820,341	34,644,955	99.97	7.5	71.63
HammerExpanded	1 h 03 m	23,197,521	2,820,316	20,377,205	99.97	12.1	76.54
BayesHammer	1 h 09 m	22,457,509	2,820,311	19,637,198	99.97	12.6	76.60

**Figure 6:**
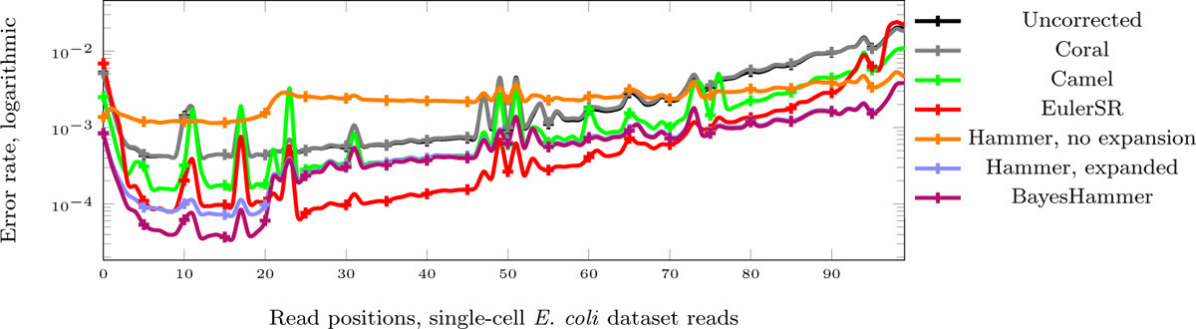
**Error reduction**. Error reduction by read position on logarithmic scale for the single-cell *E. coli*, single-cell *S. aureus*, and multi-cell *E. coli *datasets.

For a comparison of BAYESHAMMER with other tools in terms of error rate reduction across an average read, see the logarithmic error rate graphs on Figure 6. Note that we are able to count errors only for the reads that actually aligned to the genome, so the graphs are biased in this way. Note how the first 21 bases are corrected better than others in BAYESHAMMER and both versions of HAMMER since we have run it with *k *= 21; still, other values of *k *did not show a significant improvement in either *k*-mer statistics or, more importantly, assembly results.

### Assembly results

Tables 2 and 3 shows assembly results by the recently developed SPAdes assembler [10]; SPAdes was designed specifically for single-cell assembly, but has by now demonstrated state-of-the-art results on multi-cell datasets as well.

**Table 2:** Assembly results, single-cell *E.coli *and S. *aureus *datasets (contigs of length ≥ 200 are used).

Statistics	BayesHammer	BayesHammer(scaff old)	Coral	Coral (scaff old)	EulerSR	EulerSR (scaff old)	Hammer, expanded	Hammer, no expansion	Hammer, no expansion(scaff old)	Hammer(scaff old)
	**Single-cell *E. coli***, reference length 4639675, reference GC content 50.79%									

# contigs (1000 bp)	191	158	276	224	231	150	195	282	242	173
# contigs	521	462	675	592	578	375	529	655	592	477
Largest contig	269177	284968	179022	179022	267676	267676	268464	210850	210850	268464
Total length	4952297	4989404	5064570	4817757	4817757	4902434	4977294	5097148	5340871	5005022
N50	110539	113056	45672	67849	74139	95704	97639	65415	84893	109826
NG50	112065	118432	55073	87317	77762	108976	101871	68595	96600	112161
NA50	110539	113056	45672	67765	74139	95704	97639	65415	84841	109826
NGA50	112064	118432	55073	87317	77762	108976	101871	68594	96361	112161
# misassemblies	4	6	9	12	6	8	4	4	7	7
# misassembled contigs	4	6	9	10	6	8	4	4	7	7
Misass. contigs length	42496	94172	62114	150232	47372	149639	43304	26872	147140	130706
Genome covered (%)	96.320	96.315	96.623	96.646	95.337	95.231	96.287	96.247	96.228	96.281
GC (%)	49.70	49.69	49.61	49.56	49.90	49.74	49.68	49.64	49.60	49.68
# mismatches/100 kbp	11.22	11.70	8.36	9.10	5.55	5.82	12.77	54.11	52.48	13.08
# indels/100 kbp	1.07	8.26	9.17	12.76	0.52	47.80	0.91	1.17	7.96	8.69
# genes	4065 +	4079 +	3998 +	4040 +	3992 +	4020 +	4068 +	4034 +	4048 +	4078 +
	124 part	110 part	180 part	143 part	140 part	107 part	123 part	152 part	136 part	111 part

	**Single-cell *S. aureus***, reference length 2872769, reference GC content 32.75%									

# contigs (1000 bp)	95	85	132	113	82	70	114	272	258	101
Total length (1000 bp)	3019597	3309342	3055585	3066662	2972925	2993100	3033912	3389846	3405223	3509555
# contigs	260	241	455	423	166	134	312	721	711	292
Largest contig	282558	328686	208166	208166	254085	535477	282558	148002	166053	328679
Total length	3081173	3368034	3160497	3166169	3008746	3020256	3111423	3575679	3594468	3584266
N50	87684	145466	62429	90701	101836	145466	74715	30788	34943	131272
NG50	112566	194902	87636	99341	108151	159555	88292	39768	45889	180022
NA50	87684	145466	62429	89365	100509	145466	68711	30788	34552	112801
NGA50	88246	148064	74452	90101	101836	145466	88289	35998	42642	148023
# misassemblies	15	17	11	14	4	5	11	14	18	14
# misassembled contigs	12	14	9	10	4	5	9	14	16	12
Misass. contigs length	340603	779785	478009	523596	377133	918380	402997	272677	324361	940356
Genome covered (%)	99.522	99.483	99.449	99.447	99.213	99.254	99.204	98.820	98.888	99.221
GC (%)	32.67	32.63	32.64	32.63	32.66	32.67	32.67	32.39	32.38	32.57
# mismatches per 100 kbp	3.18	8.01	12.44	12.65	9.72	10.28	17.38	54.92	55.50	15.36
# indels per 100 kbp	2.17	2.30	15.50	15.67	3.80	4.08	3.57	2.64	2.72	3.04
# genes	2540 +	2547 +	2532 +	2540 +	2547 +	2550 +	2535 +	2477 +	2485 +	2539 +
	36 part	30 part	45 part	37 part	30 part	27 part	41 part	91 part	85 part	38 part

**Table 3:** Assembly results, multi-cell *E.coli *dataset (contigs of length ≥ 200 are used).

Statistics	BayesHammer	BayesHammer (sca_old)	Hammer, expanded	Hammer, no expansion	Hammer, no expansion (sca_old)	Hammer (sca_old)	Quake
	**Multi-cell E. coli**, 600 coverage, reference length 4639675, reference GC content 50.79%						

# contigs (≥ 500 bp)	103	102	119	238	213	115	165
# contigs (≥ 1000 bp)	91	90	99	192	171	96	156
Total length (≥ 500 bp)	4641845	4641790	4626515	4730338	4817457	4627067	4543682
Total length (≥ 1000 bp)	4633361	4633306	4611745	4696966	4787210	4612838	4537565
# contigs	122	121	146	325	303	141	204
Largest contig	285113	285113	218217	210240	210240	218217	165487
Total length	4647325	4647270	4635156	4756088	4844208	4635349	4555015
N50	132645	132645	113608	59167	73113	113608	58777
NG50	132645	132645	113608	59669	80085	113608	57174
NA50	132645	132645	113608	59167	73113	113608	58777
NGA50	132645	132645	113608	59669	80085	113608	57174
# misassemblies	3	3	4	4	7	5	0
# misassembled contigs	3	3	4	4	7	5	0
Misassembled contigs length	44466	44466	57908	15259	30901	60418	0
Genome covered (%)	99.440	99.440	99.383	98.891	98.925	99.385	98.747
GC (%)	50.78	50.77	50.77	50.73	50.71	50.77	50.75
N's (%)	0.00000	0.00000	0.00000	0.00000	0.00000	0.00000	0.00000
# mismatches per 100 kbp	8.55	8.55	13.76	44.46	44.33	13.76	1.21
# indels per 100 kbp	0.99	0.99	1.14	0.76	0.97	1.14	0.20
# genes	4254+45 part	4254+45 part	4245+56 part	4196+72 part	4204+68 part	4245+56 part	4174+62 part

In the tables, N50 is such length that contigs of that length or longer comprise $\geq \frac{1}{2}$ of the assembly; NG50 is a metric similar to N50 but only taking into account contigs comprising (and aligning to) the reference genome; NA50 is a metric similar to N50 after breaking up misassembled contigs by their misassemblies. NGx and NAx metrics have a more direct relevance to assembly quality than regular Nx metrics; our result tables have been produced by the recently developed tool QUAST [14].

All assemblies have been done with SPADES. The results show that after BAYESHAMMER correction, assembly results improve significantly, especially in the single-cell *E. coli *case; it is especially interesting to note that even in the multi-cell case, where BAYESHAMMER loses to QUAKE by *k*-mer statistics, assembly results actually improve over assemblies produced from QUAKE-corrected reads (including genome coverage and the number of genes).

## Conclusions

Single-cell sequencing presents novel challenges to error correction tools. In contrast to multi-cell datasets, for single-cell datasets, there is no pretty distribution of *k*-mer multiplicities; one therefore has to work with *k*-mers on a one-by-one basis, considering each cluster of *k*-mers separately. In this work, we further developed the ideas of HAMMER from a Bayesian clustering perspective and presented a new tool BAYESHAMMER that makes them practical and yields significant improvements over existing error correction tools.

There is further work to be done to make our underlying models closer to real life; for instance, one could learn a non-uniform distribution of single nucleotide errors and plug it in our likelihood formulas. Another natural improvement would be to try and rid the results of contamination by either human or some other DNA material; we observed significant human DNA contamination in our single-cell dataset, so weeding it out might yield a significant improvement. Finally, a new general approach that we are going to try in our further work deals with the technique of *minimizers *introduced by Roberts *et al*. [15]. It may provide significant reduction in memory requirements and a possible approach to dealing with paired information.

## Competing interests

The authors declare that they have no competing interests.

## Authors' contributions

All authors contributed extensively to the work presented in this paper.

## Declarations

The publication costs for this article were funded by the Government of the Russian Federation, grant 11.G34.31.0018.

This article has been published as part of *BMC Genomics *Volume 14 Supplement 1, 2013: Selected articles from the Eleventh Asia Pacific Bioinformatics Conference (APBC 2013): Genomics. The full contents of the supplement are available online at http://www.biomedcentral.com/bmcgenomics/supplements/14/S1.
